# Living alone predicts mortality in patients with ischemic stroke before 70 years of age: a long-term prospective follow-up study

**DOI:** 10.1186/s12883-016-0599-y

**Published:** 2016-05-27

**Authors:** Petra Redfors, Daniella Isaksén, Georgios Lappas, Christian Blomstrand, Annika Rosengren, Katarina Jood, Christina Jern

**Affiliations:** Institute of Neuroscience and Physiology, the Sahlgrenska Academy at University of Gothenburg, Gothenburg, Sweden; Institute of Biomedicine, the Sahlgrenska Academy at University of Gothenburg, Gothenburg, Sweden; Institute of Medicine, the Sahlgrenska Academy at University of Gothenburg, Gothenburg, Sweden

**Keywords:** Cerebral infarction, Mortality, Prognosis, Socioeconomic factors, Stroke subtypes, Follow-up studies

## Abstract

**Background:**

Living alone is associated with increased mortality after myocardial infarction but little data is available about whether this applies to prognosis after stroke. We aimed to examine the association between living situation and long-term mortality in patients with ischemic stroke and a control group, and to explore whether this association is modified by patient gender.

**Methods:**

This is a prospective case-control study of 600 patients with ischemic stroke before 70 years of age and 600 age- and sex-matched controls who have been included in the Sahlgrenska Study on Ischemic Stroke. Mortality data were collected through national registers and medical records. We used Cox regression models for identifying predictors of mortality.

**Results:**

In the entire sample, mean age was 57 years, proportion of males 64 %, proportion living alone 28 %, and median follow-up 8.6 years. Mortality rates were 36 % among patients living alone, 17 % among cohabitant patients, 15 % among controls living alone, and 9 % among cohabitant controls. Living alone was an independent predictor of all-cause mortality in cases after adjustment for stroke severity, stroke subtype, and vascular risk factors including physical activity, alcohol consumption, and socioeconomic status. A significant interaction was found between gender and living situation; the adjusted hazard ratio for mortality was 3.47 (95 % Confidence Interval 2.13–5.65) in male patients living alone, whereas no significant association was observed in women. Living alone was also a predictor of vascular mortality among cases and of all-cause mortality among controls.

**Conclusions:**

Living alone is associated with increased long-term mortality after ischemic stroke in men. Further prospective studies are needed to confirm the observed gender difference and to identify modifiable factors underlying this increased risk.

**Electronic supplementary material:**

The online version of this article (doi:10.1186/s12883-016-0599-y) contains supplementary material, which is available to authorized users.

## Background

Living arrangements have changed markedly in recent decades [1, 2]. In many Western countries as many as 30 % of the population are now living alone, and the proportion of middle-aged people living alone has increased quite dramatically. Marriage has long been known to be associated with reduced mortality [3], but this protective effect seems to be attenuated at older ages [2, 4, 5]. Studies that have specifically investigated living situation (i.e. cohabitant status) have found a strong association with mortality [6, 7]. With regard to vascular disease, a recent large register study from Finland shows that living situation is a stronger determinant for long-term fatality after myocardial infarction (MI) than for incidence of MI [1]. This study and a community-based study from Canada indicate that the increased mortality after MI in patients living alone is restricted to males [1, 7], findings that are in line with studies showing that for general mortality, the protective effect of marriage is stronger in men compared to women [2, 4, 5, 8].

Evidence is scarce regarding living alone as a predictor of mortality after stroke. Waje-Andreassen et al. prospectively followed 232 young patients with ischemic stroke and found that living alone was a predictor of long-term mortality [9]. The Manhattan stroke study identified social isolation, but not living alone, as a predictor of a combined outcome of vascular events and/or death [10]. In a more recent study based on Riks-Stroke, living alone was an independent predictor of short-term post-stroke mortality [11]. In this study the association between living situation and one-year mortality was only significant in men. Furthermore, the difference in mortality related to living situation increased during the one-year follow-up, and it was less pronounced in older age groups. These findings motivate studies of young and middle-aged patients using a more long-term follow-up period. Moreover, initial stroke severity is a well-recognized predictor of post-stroke mortality, and a recent study suggests that stroke severity is associated with living situation [12]. Thus, it may also be of importance to take into consideration the possible influence of stroke severity on the association between living situation and post-stroke mortality.

The current aim was to examine the association between living situation and mortality by conducting a very long-term follow-up of young and middle-aged ischemic stroke patients and controls, and to determine whether this association was independent of initial stroke severity and stroke subtype, as well as vascular risk factors and other socioeconomic factors. Because prior studies have suggested that men may be more susceptible to the effects of living alone [2, 4, 11], we also wanted to explore whether this association was modified by patient gender.

## Methods

### Study design and baseline data

The sample comprised participants from the Sahlgrenska Academy Study on Ischemic Stroke (SAHLSIS), which has been described in detail elsewhere [13]. In brief, it included 600 patients with ischemic stroke consecutively recruited at four stroke units in western Sweden between 1998 and 2003, and 600 controls randomly selected to match the cases with regard to age, gender and geographical area. SAHLSIS included patients below 70 years of age, which allowed for long-term follow-up. In view of inclusion being hospital-based, it is worth noting that according to the Swedish National Guidelines for Stroke Care from 2005, all patients with suspected stroke should be hospitalized in a stroke unit regardless of stroke severity. Even before these guidelines were launched in Sweden, more than 95 % of patients with suspected stroke before 74 years of age were hospitalized [14].

Stroke severity was assessed using the Scandinavian Stroke Scale (SSS). Etiologic stroke subtype was classified according to the Trial of Org 10172 in Acute Stroke Treatment (TOAST) system. Cryptogenic and undetermined stroke were analyzed as separate groups and were defined according to the following criteria: no cause identified after a broad evaluation had been done and when more than one cause was identified or when the evaluation was insufficient, respectively. For more details on stroke subtyping, see Additional file 1.

Living situation was categorized as living with a partner or an adult family member (sibling or child over 20 years of age or parent) versus living alone at the time of index stroke for cases and at baseline for controls. Information on living situation and vascular risk factors was obtained from structured questionnaires, an interview, and examinations at inclusion. Definitions of vascular risk factors, comorbidities, occupational classification, leisure-time physical activity, and self-perceived psychological stress have been described elsewhere [13, 15, 16]. The definitions of waist-hip-ratio categories, alcohol consumption, pre-stroke disability, and personal history of coronary heart disease are given in the Additional file 1.

### Follow-up and outcomes

By using the unique 10-digit Swedish person identity number, all participants who had died by the 31^st^ of December 2010 were identified. Information on the cause of death was obtained from the Swedish Cause of Death Register, which is based on the International Classification of Diseases, 10^th^ Revision (ICD10). We also reviewed medical records within 6 months prior to death, both for participants who died at hospital and who died at home. Causes of death were categorized into vascular deaths and non-vascular deaths. For a detailed description of the collection and classification of data on death see the on-line Additional file 1.

### Statistical analysis

Comparisons between living situation groups were examined with the χ^2^-test or Fisher’s exact test as appropriate for the categorical variables and with the Students *t*-test for the continuous variables. The survival function and corresponding 95 % confidence intervals (CI) for the follow-up period were estimated according to the Kaplan-Meier method.

Univariable Cox regression analyses for all-cause and vascular mortality were performed for age, gender, living situation, vascular risk factors, socioeconomic factors, stroke severity, and stroke subtype. As SSS scores were skewed and could not be transformed to normal distribution, stroke severity was classified as severe (0–25), moderate (26–42) and mild (43–58) [17]. Occupational classification, physical activity, alcohol consumption, psychological stress, and smoking were considered as possible confounding factors regardless of *P* value in the univariable analyses. In the multivariable models, the impact of living situation for all-cause and vascular mortality was analyzed by creating Cox regression models where we included the predefined confounding factors as well as variables with *P* < 0.1 in the univariable analyses. Missing values concerning the categorical variables were included as dummy variables. In the multivariable analysis of all-cause mortality there was a significant interaction between gender and living situation, and thus men and women were entered separately into this model. The assumption of proportional hazards was checked for each covariate through the cumulative sums of martingale residuals (ASSESS statement in the PROC PHREG analytical procedure in SAS statistical package 9.3, which estimates parameters in a Cox regression model procedure). To maintain the assumption of proportional hazards, the effects of sedentary leisure time, personal history of coronary heart disease [18] and the stroke subtype cryptogenic stroke were entered into the multivariable model for all-cause mortality as time-dependent variables, as these variables displayed a varying influence on mortality during the follow-up period.

## Results

The mean age for all participants at inclusion was 57 years, and 64 % were males. All patients were followed for a minimum of 7.0 years or until death, and their median time of follow-up was 8.8 years (interquartile range (IQR) 7.7-10.0), corresponding to 5,080 person years in total. Median follow-up time for controls was 8.2 years (IQR 7.7-10.0), in total 5,199 person years. No patient or control was lost to follow-up. However, four participants moved abroad, but were followed for a minimum of 4.4 years.

Of the 600 patients, 176 (29.3 %) were living alone at baseline, and the proportion of controls living alone was similar (*n* = 163; 27.2 %). Table 1 shows the baseline characteristics for patients according to living situation. Patients living alone were more likely to be smokers, to have an occupation with lower educational level, to have sedentary leisure-time, and pre-stroke disability. Baseline characteristics for controls according to living situation are shown in Additional file 1: Table S1.

**Table 1:** Baseline characteristics for cases stratified by living situation^a^

	Cases		
	Cohabiting (*n*=424)	Living alone (*n*=176)	*P*
Mean age at inclusion, years	57 (10)	56 (11)	0.37
Male	278 (66)	107 (61)	0.27
Risk factors			
Hypertension	250 (59)	104 (61)	0.66
Diabetes mellitus	81 (19)	33 (19)	0.92
Hyperlipidemia	293 (75)	120 (79)	0.28
Waist-hip-ratio, gender-adjusted^b^			
Low	4 (1)	4 (2)	0.37
Normal	87 (22)	31 (19)	
Intermediate	111 (28)	41 (25)	
High	187 (48)	85 (53)	
Smoking	141 (33)	92 (53)	<0.001
Socioeconomic and life-style factors			
Occupation, lower education	242 (59)	122 (73)	0.001
Sedentary leisure time	60 (15)	48 (30)	<0.001
Self-perceived psychological stress	92 (23)	34 (21)	0.64
Alcohol consumption >4 times a week	27 (7)	17 (10)	0.14
Pre-stroke disability	2 (0.5)	7 (4)	<0.01
Comorbidities			
History of stroke	85 (20)	29 (16)	0.80
History of coronary heart disease	71 (18)	26 (17)	0.85
Atrial fibrillation	52 (12)	16 (9)	0.82
Endovascular treatment	4 (0.9)	1 (0.6)	1.00
Stroke severity			
Mild, SSS score 43-58	321 (76)	130 (74)	0.81
Moderate, SSS score 26-42	63 (15)	30 (17)	
Severe, SSS score 0-25	40 (9)	16 (9)	
Stroke subtype			
Small vessel disease	81 (19)	43 (24)	0.63
Large vessel disease	52 (12)	21 (12)	
Cardioembolic stroke	72 (17)	26 (15)	
Cryptogenic stroke	112 (26)	50 (28)	
Other determined stroke	38 (9)	13 (7)	
Undetermined stroke	69 (16)	23 (13)	

*SSS* Scandinavian Stroke Scale

^a^Data are no. (%) unless otherwise indicated

^b^Waist-hip-ratio was categorized as low, normal (reference), moderate and high; gender-specific cut-offs (for men: <0.85, 0.85-<0.95, 0.95-<1, and ≥1, and for women: <0.7, 0.7-<0.8, 0.8-<0.85, and ≥0.85)

During the follow-up period, 112 (18.7 %) patients and 34 (5.7 %) controls died, which corresponds to 22.0 and 6.5 deaths per 1,000 person years, respectively (log rank *P* < 0.001). The cumulative rates for all-cause mortality for patients were 2.0 % (95 % CI 0.8–3.2 %) at 1 year, 9.5 % (95 % CI 7.1–11.9 %) at 5 years, and 20.2 % (95 % CI 16.7–23.7 %) at 10 years. For controls these rates were 0 % at 1 year, 2.2 % (95 % CI 1.0–3.4 %) at 5 years, and 8.0 % (95 % CI 4.9–11.1 %) at 10 years.

**Fig. 1:**
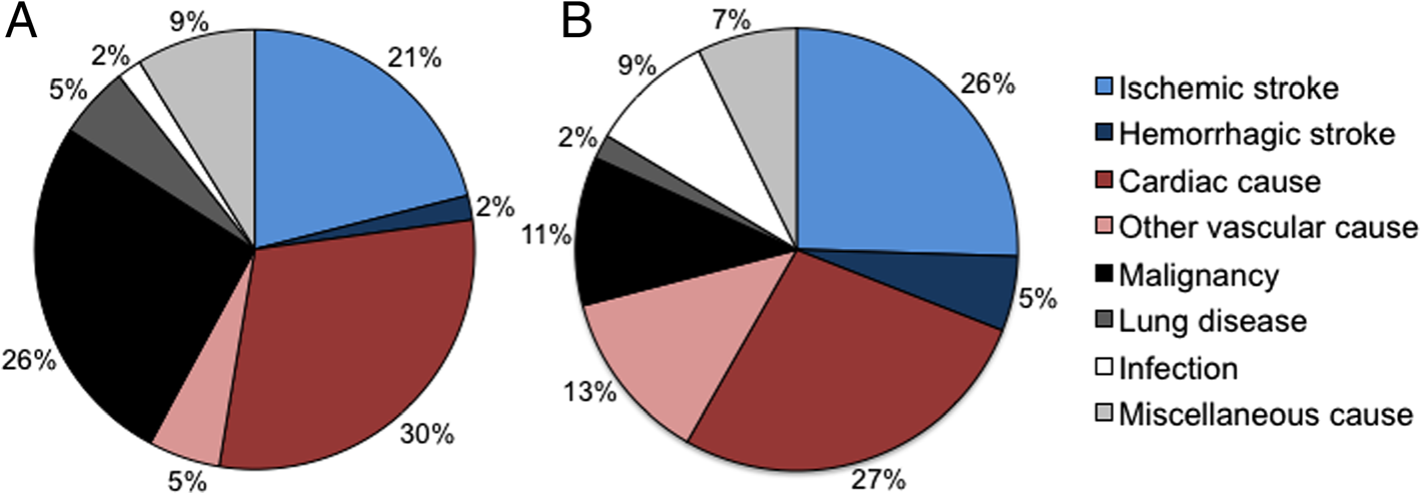
Causes of death for cohabitant patients (Panel **a**) and for patients living alone (Panel **b**)

The increased mortality for patients was mainly due to vascular causes and lung cancer. Seventy-two (64.3 %) deaths among patients and 9 (26.5 %) deaths among controls were due to vascular causes. The proportion of cancer deaths that was due to lung cancer was 52.4 % and 25.0 % for cases and controls, respectively. Figure 1 displays the causes of death in patients according to living situation. The proportion of deaths due to vascular causes was greater in patients living alone compared to cohabitant patients (*P* < 0.001). With regard to miscellaneous causes, three patients committed suicide, one of whom was living alone before the index stroke. The proportion of deaths due to vascular causes was 33.3 % in controls living alone and 21.1 % in cohabitant controls.

**Fig. 2:**
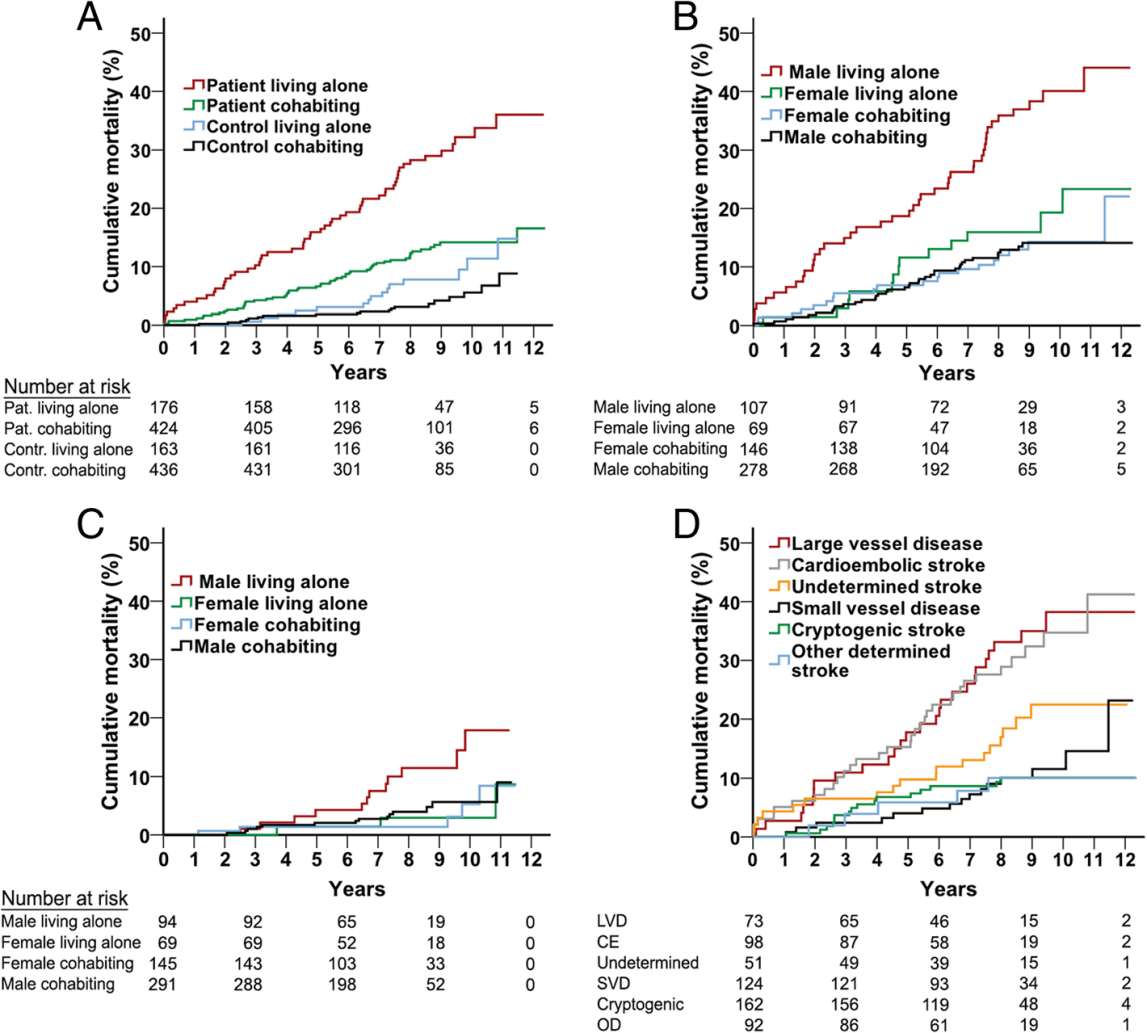
Cumulative mortality rates according to living situation for patients and controls (Panel **a**), for patients according to living situation and gender (Panel **b**), for controls according to living situation and gender (Panel **c**), and for patients according to etiologic stroke subtype (Panel **d**). Abbreviations; Pat., patients; Contr., controls; LVD, large vessel disease; SVD, small vessel disease; CE, cardioembolic; and OD, other determined

Subjects living alone had a higher mortality rate compared to cohabiting subjects throughout the follow-up period. The cumulative mortality rate was 36.1 % among patients living alone compared to 16.6 % among cohabiting patients and 14.8 % among controls living alone compared to 8.8 % among cohabiting controls (log rank *P* < 0.001, Fig. 2a). For male and female patients living alone, the mortality rates were 44.1 % and 23.3 %, respectively, compared to 14.1 % for cohabiting male patients and 22.1 % for cohabiting female patients (log rank *P* < 0.001, Fig. 2b). A similar gender pattern was seen in controls (log rank *P* = 0.007, Fig. 2c). As expected, the mortality rate also varied by stroke subtype (log rank *P* < 0.001, Fig. 2d).

Table 2 presents crude and adjusted associations between baseline characteristics and all-cause mortality in patients. In the multivariable Cox regression model there was an interaction between gender and living situation (*P* = 0.02). Among male patients, living alone was an independent predictor of all-cause mortality, whereas no significant association was detected for female patients. When restricting the analysis to vascular mortality, living alone remained as an independent predictor of mortality in the entire group, but there was no significant interaction between gender and living situation (Additional file 1: Table S2).

**Table 2:** Cox regression analyses investigating predictors of all-cause mortality for cases (*n* = 600)

	All-cause mortality					
	Univariable			Multivariable		
	HR	95 % CI	*P*	HR	95 % CI	*P* ^a^
Age	1.05	(1.03–1.08)	<0.001	1.05	(1.02–1.08)	<0.001
Male	1.38	(0.92–2.07)	0.12	0.80	(0.45–1.41)	0.44
Living alone, females	1.38	(0.68–2.77)	0.38	1.28	(0.62–2.64)	0.50
Living alone, males	3.33	(2.14–5.18)	<0.001	3.47	(2.13–5.65)	<0.001
Gender-living alone	3.11	(2.12–4.56)	<0.001	2.70	(1.14–6.43)	0.02
Risk factors						
Hypertension	1.16	(0.78–1.71)	0.47	-	-	
Diabetes mellitus	2.32	(1.56–3.44)	<0.001	2.10	(1.36–3.24)	<0.001
Hyperlipidemia	1.01	(0.63–1.64)	0.96	-	-	
Smoking	1.62	(1.11–2.35)	0.01	1.31	(0.87–1.96)	0.20
Socioeconomic and life-style factors						
Occupation, lower education	1.39	(0.91–2.11)	0.13	1.07	(0.68–1.68)	0.79
Sedentary leisure time^b^	1.86	(1.22–2.84)	0.006	0.76^c^	(0.36–1.63)	0.48
				1.51^d^	(0.87–2.63)	0.14
Self-perceived psychological stress	0.79	(0.48–1.30)	0.35	0.99	(0.58–1.72)	0.98
Alcohol consumption >4 times a week	1.61	(0.88–2.94)	0.12	1.41	(0.74–2.68)	0.30
Comorbidities						
History of coronary heart disease^b^	2.64	(1.73–4.04)	<0.001	0.80^c^	(0.36–1.80)	0.60
				2.33^d^	(1.34–4.05)	0.003
History of stroke	1.19	0.76–1.86)	0.46	-	-	
Stroke severity						
Mild, SSS score 43–58	Reference			Reference		
Moderate, SSS score 26–42	1.94	(1.25–3.01)	0.003	1.63	(1.03–2.58)	0.04
Severe, SSS score 0–25	1.30	(0.69–2.45)	0.42	0.99	(0.51–1.95)	0.99
TOAST subtype						
Small vessel disease	Reference			Reference		
Large vessel disease	3.33	(1.77–6.30)	<0.001	2.75	(1.42–5.33)	0.003
Cardioembolic stroke	3.15	(1.70–5.82)	<0.001	2.80	(1.44–5.42)	0.003
Cryptogenic stroke^b^	0.81	(0.40–1.64)	0.56	2.18^c^	(0.94–5.07)	0.07
				0.58^d^	(0.20–1.63)	0.30
Other determined stroke	0.79	(0.29–2.18)	0.65	1.58	(0.55–4.56)	0.40
Undetermined stroke	1.75	(0.88–3.48)	0.11	1.94	(0.96–3.93)	0.07

*HR* hazard ratio, *CI* confidence interval, *SSS* Scandinavian Stroke Scale

^a^Adjusted for age, sex, living situation, diabetes mellitus, smoking, occupation, leisure physical activity, self-perceived psychological stress, alcohol consumption, history of coronary heart disease, stroke severity, and stroke subtype

^b^Time-dependent variable was used

^c^For 0–4 years

^d^For 4–8 years

Living alone was a predictor for all-cause mortality also among controls (hazard ratio (HR) 2.45, 95 % CI 1.23–4.86; *P* = 0.011). This association was independent of age and gender, but due to the low number of deaths in this group, further adjustments were not done.

## Discussion

In this longitudinal study of young and middle-aged patients with ischemic stroke living alone was as an independent predictor of long-term mortality. The unique contribution of this study includes a long and complete follow-up and a well-characterized cohort that allowed us to account for a range of covariates including important potential confounding/mediating factors. Our study also included a population-based control group with a mortality rate that was less than half of that of cases.

The finding that living alone predicts long-term post-stroke mortality is in line with the results presented by Waje-Andreassen et al. [9]. In addition, our results also show that the increased mortality among patients living alone is mainly due to vascular causes, and that the association between living situation and long-term mortality is independent of vascular risk factors, other socioeconomic factors as well as stroke severity and subtype. Furthermore, we observed more deaths from infections among patients living alone, but this finding was based on small numbers.

We also found a significant interaction between gender and living situation, and male patients living alone were twice as likely to die as female patients living alone. This finding is supported by recent data on 1-year post-stroke mortality from Riks-Stroke [11] and by studies on mortality after MI [1, 7]. Consistent with population-based studies, we also observed a gender-specific difference in mortality among controls living alone [2, 4].

The mechanisms by which living alone can affect long-term post-stroke mortality remain to be determined. Living alone may be viewed as a proxy for low social support, and for coronary heart disease, biological mechanisms such as proinflammatory and prothrombotic states and psychological distress have been proposed [19, 20]. Moreover, health-compromising behaviors such as smoking, heavy drinking, physical inactivity, less frequent dental visits, and impaired medication adherence have been associated with living without a partner [19, 21], and are thus likely to mediate some of the effect, although some of these variables were accounted for in the present study. There are many potential mechanisms that may explain the gender-specific difference in mortality among stroke patients living alone. As a couple of examples, men appear to be more dependent on the encouragement of their spouses to seek medical attention for cardiovascular symptoms compared to women [22], and men living alone has a higher likelihood of having an unhealthy diet compared to women living alone and cohabitants persons [23].

Regarding other predictors of post-stroke outcomes, we found that the stroke subtypes of large vessel disease and cardioembolic stroke, as well as diabetes mellitus, were independent predictors of long-term mortality, in similarity with other studies [24, 25]. Stroke severity has been recognized as a predictor for long-term mortality [26], but in the present study an increased mortality was only detected for the group with moderate stroke severity. However, the lack of an association with severe stroke is most likely explained by the low number of patients in this group in the age group under study here. It is also of note that in contrast to a recent study from US (12), we did not observe any association between living situation and stroke severity. A possible explanation may be differences in case mix.

Strengths of the present study include the large and well-characterized sample of consecutively recruited ischemic stroke cases with complete long-term follow-up and with detailed information on cause of death. There are also some limitations to be considered. Biases in selection of subjects may occur in case-control design. However, in Sweden, practically all patients with stroke are hospitalized, regardless of stroke severity, and the early case fatality for the age group under study is low. Thus, as expected, cumulative mortality rates in the present cohort was similar compared to population-based studies [26, 27]. Living situation may change during the follow-up period, but 7-year follow-up data for surviving patients in the present study showed that only a very minor proportion had changed living situation (see Additional file 1). Depression is increasingly recognized as associated with mortality after stroke [28], but we lacked data on depression at baseline. However, according to a literature review, depression and low social support are independent predictors of poor outcomes after coronary heart disease [29]. We had no information on non-prescribed narcotics, but no patient was hospitalized due to drug abuse during the 7-year follow-up. Finally, we had no data on medication during follow-up and thus cannot exclude that differences in prescription have influenced the results, although treatment compliance probably has a greater impact as discussed above.

## Conclusion

In conclusion, living alone predicts long-term mortality after ischemic stroke in young and middle-aged subjects, and the association is independent of vascular risk factors and several other socioeconomic factors as well as stroke severity and subtype. In line with existing evidence after MI, this association was confined to men. Further studies are required to confirm the gender-specific influence, and to identify underlying mechanisms.

## Additional file

Additional file 1: Supporting information. **Table S1.** Baseline 368 characteristics for cases and controls, and stratified by living situation for 369 controls. **Table S2.** Cox regression analyses investigating predictors of 370 vascular mortality for cases. (DOCX 171 kb)

## Abbreviations

CI: Confidence interval
HR: Hazard ratio
ICD10: International classification of diseases 10^th^ revision
IQR: Interquartile range
MI: Myocardial infarction
SAHLSIS: Sahlgrenska Academy Study on Ischemic Stroke
TOAST: Trial of Org 10172 in Acute Stroke Treatment
