# Supplementary material for: Living alone predicts mortality in patients with ischemic stroke before 70 years of age: a long-term prospective follow-up study
Source: BMC Neurol. 2016 May 27;16:80. doi: 10.1186/s12883-016-0599-y (PMC4942912; doi:10.1186/s12883-016-0599-y)
Supplement: Additional file 1: — Supporting information. Table S1. Baseline 368 characteristics for cases and controls, and stratified by living situation for 369 controls. Table S2. Cox regression analyses investigating predictors of 370 vascular mortality for cases. (DOCX 171 kb) [file 12883_2016_599_MOESM1_ESM.docx]

# Additional file 1

## Supplemental Methods

All patients underwent standard investigations at the stroke unit, which in every case included computed tomography of the brain. Magnetic resonance imaging of the brain was performed in 62% of the patients.

### Baseline data

Waist-hip-ratio was categorized as low, normal (reference), moderate and high with gender-specific cut-offs as defined by World Health Organization (for men: <0.85, 0.85-<0.95, 0.95-<1 and ≥1, and for women: <0.7, 0.7-<0.8, 0.8-<0.85 and ≥0.85) [[1](#_ENREF_1)]. Alcohol consumption was categorized as alcohol consumption ≥4 times a week versus <4 times a week or never, based on self-assessment at inclusion. Pre-stroke disability was defined as receiving health-related home help (including paid care) or living at a nursing home prior to the stroke event. Personal history of coronary heart disease was defined as having suffered a myocardial infarction (MI) or having ECG changes indicating previous MI. All patients were classified into etiologic subtypes of ischemic stroke according to the Trial of Org 10172 in Acute Stroke Treatment (TOAST) system. The main etiological subtypes were large vessel disease, small vessel disease, and cardioembolic stroke. Stroke was classified as cryptogenic when no cause was identified despite an extensive evaluation. Undetermined stroke included cases in which more than one cause was identified or when the evaluation was insufficient. Ascertainment of subtypes were performed by two neurologists (KJ and CB).

In order to minimize interrater variability, the original TOAST criteria were refined according to a local protocol, which has been described in detail elsewhere [[2](#_ENREF_2)].

### Collection of follow-up data on death

For all participants, the Swedish Hospital Discharge register for all admissions within 6 months before death and relevant medical records were reviewed. The review included neuroimaging and relevant laboratory data.

In 20 cases the cause of death initially identified in the Swedish Cause of Death register was changed after a thorough examination of the medical records. Twenty-six (23%) patients and 5 (15%) controls were autopsied. In 57 (51%) patients and 16 (47%) controls, sufficient information on cause of death was present in the medical records. For the remainder, cause of death was obtained from the Swedish Cause of Death Register.

### Classification of non-vascular and vascular death

For vascular death the following ICD-10 codes were used: I10-I28, I42-I50, I60-I89 and F01. Death was also considered to be vascular if occurring within 30 days from a major stroke or an MI. Vascular death was further classified into ischemic stroke, hemorrhagic stroke, cardiac causes and other vascular causes. Cardiac causes included coronary disease, cardiomyopathy and congestive heart failure. Other vascular causes were, for example, aortic dissection and pulmonary embolus unless secondary to surgery or under the course of malignant disease. Non-vascular causes were malignancies, infections, lung diseases and miscellaneous causes such as trauma, suicide and liver failure.

## Supplemental Results

### Living situation in follow-up

For patients, we also checked living situation 3 months after index stroke. Two patients (0.3%) had divorced and 1 (0.2%) had been temporarily cohabitant with his ex-wife. At 7 years, 439 patients were eligible for follow-up questionnaires, among which 41 (9.3%) had changed cohabitation status, 18 (4.1%) had become widows / widowers, 18 (4.1%) had divorced and 5 (1.1%) had begun cohabiting

1.Hotchkiss JW, Davies CA, Leyland AH. Adiposity has differing associations with incident coronary heart disease and mortality in the Scottish population: cross-sectional surveys with follow-up. Int J Obes (Lond). 2013;37(5):732-739.

2.Olsson S, Holmegaard L, Jood K, Sjogren M, Engstrom G, Lovkvist H et al. Genetic variation within the interleukin-1 gene cluster and ischemic stroke. Stroke. 2012;43(9):2278-2282.

**Table S1** Baseline characteristics for controls stratified by living

situation^1^

|  | **Controls** | | |
| --- | --- | --- | --- |
|  | **Cohabiting (n=436)** | **Living alone**  **(n=163)** | ***P*** |
| Age, mean years | 57 (9) | 55 (13) | 0.02 |
| Male | 291 (67) | 94 (58) | 0.04 |
| Risk factors |  |  |  |
| Hypertension | 168 (39) | 56 (35) | 0.37 |
| Diabetes mellitus | 24 (6) | 9 (6) | 1.00 |
| Hyperlipidemia | 311 (71) | 91 (56) | <0.001 |
| Waist-hip-ratio, gender-adjusted^2^ |  |  |  |
| Low | 7 (2) | 5 (3) | 0.22 |
| Normal | 156 (36) | 50 (31) |  |
| Intermediate | 125 (29) | 41 (25) |  |
| High | 145 (33) | 66 (41) |  |
| Smoking | 73 (17) | 35 (21) | 0.18 |
| Socioeconomic and life-style factors |  |  |  |
| Occupation, lower education | 216 (50) | 91 (59) | 0.04 |
| Sedentary leisure time | 26 (6) | 16 (10) | 0.11 |
| Self-perceived psychological stress | 27 (6) | 19 (12) | 0.03 |
| Alcohol consumption >4 times a week | 27 (6) | 16 (10) | 0.13 |
| Pre-stroke disability | - | - |  |
| Comorbidities |  |  |  |
| History of stroke | 0 | 0 |  |
| History of coronary heart disease | 0 | 0 |  |
| Atrial fibrillation | 4 (0.9) | 3 (2) | 0.35 |

^1^Data are no. (%) unless otherwise indicated. ^2^Waist-hip-ratio was

categorized as low, normal (reference), moderate and high; gender-specific

cut-offs (for men: <0.85, 0.85-<0.95, 0.95-<1, and ≥1, and for women:

<0.7, 0.7-<0.8, 0.8-<0.85, and ≥0.85).

**Table S2** Cox regression analyses investigating predictors of vascular mortality for cases (n=600)

|  | Vascular mortality | | | | | |
| --- | --- | --- | --- | --- | --- | --- |
|  | Univariable | | | Multivariable | | |
|  | HR | (95% CI) | *P* | HR | (95% CI) | *P*^1^ |
| Age | 1.09 | (1.05-1.13) | <0.001 | 1.06 | (1.02-1.11) | 0.004 |
| Male | 1.73 | (1.01-2.94) | 0.05 | 1.20 | (0.66-2.16) | 0.55 |
| Living alone | 3.06 | (1.92-4.86) | <0.001 | 3.32 | (1.94-5.68) | <0.001 |
| Risk factors |  |  |  |  |  |  |
| Hypertension | 1.75 | (1.03-2.98) | 0.04 | 1.45 | (0.79-2.66) | 0.24 |
| Diabetes | 2.19 | (1.34 -3.60) | <0.002 | 1.49 | (0.84-2.65) | 0.18 |
| Hyperlipidemia | 0.90 | (0.50-1.62) | 0.73 | - | - |  |
| Smoking | 1.28 | (0.80-2.04) | 0.31 | 1.32 | (0.88-2.00) | 0.18 |
| Socioeconomic and life-style factors |  |  |  |  |  |  |
| Occupation, lower education | 1.38 | (0.81-2.33) | 0.23 | 0.98 | (0.55-1.76) | 0.96 |
| Sedentary leisure time | 2.25 | (1.35-3.73) | 0.003 | 1.35 | (0.76-2.39) | 0.31 |
| Self-perceived  psychological stress | 0.63 | (0.32-1.23) | 0.18 | 0.88 | (0.41-1.90) | 0.75 |
| Alcohol consumption  >4 times a week | 1.72 | (0.82-3.60) | 0.15 | 2.11 | (0.92-4.85) | 0.08 |
| Comorbidities |  |  |  |  |  |  |
| History  of coronary disease | 3.31 | (2.00-5.58) | <0.001 | 1.95 | (1.08-3.53) | 0.03 |
| History  of stroke | 1.45 | (0.85-2.47) | 0.18 | - | - |  |
| Stroke severity |  |  |  |  |  |  |
| Mild, SSS score 43-58 | Reference | | | Reference | | |
| Moderate, SSS score 26-42 | 2.40 | (1.39-4.12) | 0.002 | 2.00 | (1.10-3.67) | 0.02 |
| Severe, SSS score 0-25 | 2.28 | (1.18-4.43) | 0.01 | 1.20 | (0.51-2.86) | 0.68 |
| TOAST subtype |  |  |  |  |  |  |
| Small vessel disease | Reference | | | Reference | | |
| Large vessel disease | 6.04 | (2.58-14.14) | <0.001 | 5.43 | (2.18-13.48) | <0.001 |
| Cardioembolic stroke | 5.29 | (2.29-12.22) | <0.001 | 4.42 | (1.77-11.00) | 0.001 |
| Cryptogenic stroke | 0.65 | (0.22-1.94) | 0.44 | 1.03 | (0.34-3.16) | 0.95 |
| Other determined stroke | 0.68 | (0.14-3.27) | 0.63 | 1.46 | (0.29-7.50) | 0.65 |
| Undetermined stroke | 2.09 | (0.80-5.50) | 0.13 | 1.95 | (0.71-5.36) | 0.20 |

HR, hazard ratio; CI, confidence interval; SSS, Scandinavian Stroke Scale.

^1^Adjusted for age, sex, living situation, hypertension, diabetes, smoking, occupation, leisure physical activity, self-perceived psychological stress, alcohol consumption, history of coronary disease, stroke severity, and stroke subtype.
